# Complement in Kidney Transplantation

**DOI:** 10.3389/fmed.2017.00066

**Published:** 2017-05-30

**Authors:** Marek Cernoch, Ondrej Viklicky

**Affiliations:** ^1^Transplant Laboratory, Transplant Center, Institute for Clinical and Experimental Medicine, Prague, Czechia; ^2^Department of Nephrology, Transplant Center, Institute for Clinical and Experimental Medicine, Prague, Czechia

**Keywords:** complement, kidney transplantation, antibody-mediated rejection, ischemia/reperfusion injury, anticomplement therapy

## Abstract

The complement system is considered to be an important part of innate immune system with a significant role in inflammation processes. The activation can occur through classical, alternative, or lectin pathway, resulting in the creation of anaphylatoxins C3a and C5a, possessing a vast spectrum of immune functions, and the assembly of terminal complement cascade, capable of direct cell lysis. The activation processes are tightly regulated; inappropriate activation of the complement cascade plays a significant role in many renal diseases including organ transplantation. Moreover, complement cascade is activated during ischemia/reperfusion injury processes and influences delayed graft function of kidney allografts. Interestingly, complement system has been found to play a role in both acute cellular and antibody-mediated rejections and thrombotic microangiopathy. Therefore, complement system may represent an interesting therapeutical target in kidney transplant pathologies.

## Introduction

The complement system was, for a long time since its discovery, considered to be a mere support system for the innate immunity mechanisms, playing a role in bacterial lysis. Over the years, it became obvious that the complement is an important part of the immune system, and its functions are more various than previously assumed. It was shown to play an important role in various kidney-related diseases. Its participation was observed in various forms of glomerulonephritis, including those connected to systemic diseases, for example, systemic lupus erythematodes. The complement was also found to participate in other renal diseases and conditions, such as atypical hemolytic uremic syndrome (aHUS), IgA nephropathy, or diabetes mellitus-related renal complications. The complement system was also shown to be involved in many of the transplant-related pathologies. Complement proteins also play a role in the ischemia/reperfusion injury, the innate and adaptive alloimmune responses. Recently, many complement-targeting therapies have been suggested in organ transplantation.

## Role of the Complement System

The complement system includes more than 40 proteins, both membrane-bound and soluble, including receptors and various regulatory proteins (1). The complement system is a fully integrated part of immune system, capable of cooperation with other parts of immune system (both native and adaptive) (2).

Components of the complement system function in cooperation when the complement is activated, resulting in a coordinated sequence of enzymatic activities and leading to the creation of components with effector function (3). The functions of complement system reach far beyond direct bacterial membrane lysis, which is performed by the membrane attack complex (MAC), a result of terminal complement cascade (4). The complement also participates in apoptotic cell removal (5), in a strictly controlled manner in order to prevent excessive complement activation which could lead to cell lysis and necrosis (6). Anaphylatoxins generated by C3 and C5 cleavage (i.e., C3a and C5a) are generally considered to have the pro-inflammatory effect and to possess a chemotactic capability, attracting and activating granulocytes, mast cells, and phagocytes (7). Complement components bound to the pathogen surface also opsonize the pathogen, marking it for phagocytosis (8).

The complement receptors were found to be able to influence B cell activation and the antibody production (9). Studies also showed that T cells are functioning in close cooperation with complement proteins, producing complement component endogenously. Conversely, complement anaphylatoxins C3a and C5a were shown to function as both costimulatory and survival signals to naive CD4+ T cells (10). The absence of this signaling was later found to have the effect of changing the cytokine production in CD4+ T cells, resulting in the creation of induced regulatory T cells (11).

## Complement Activation Pathways

**Figure 1:**
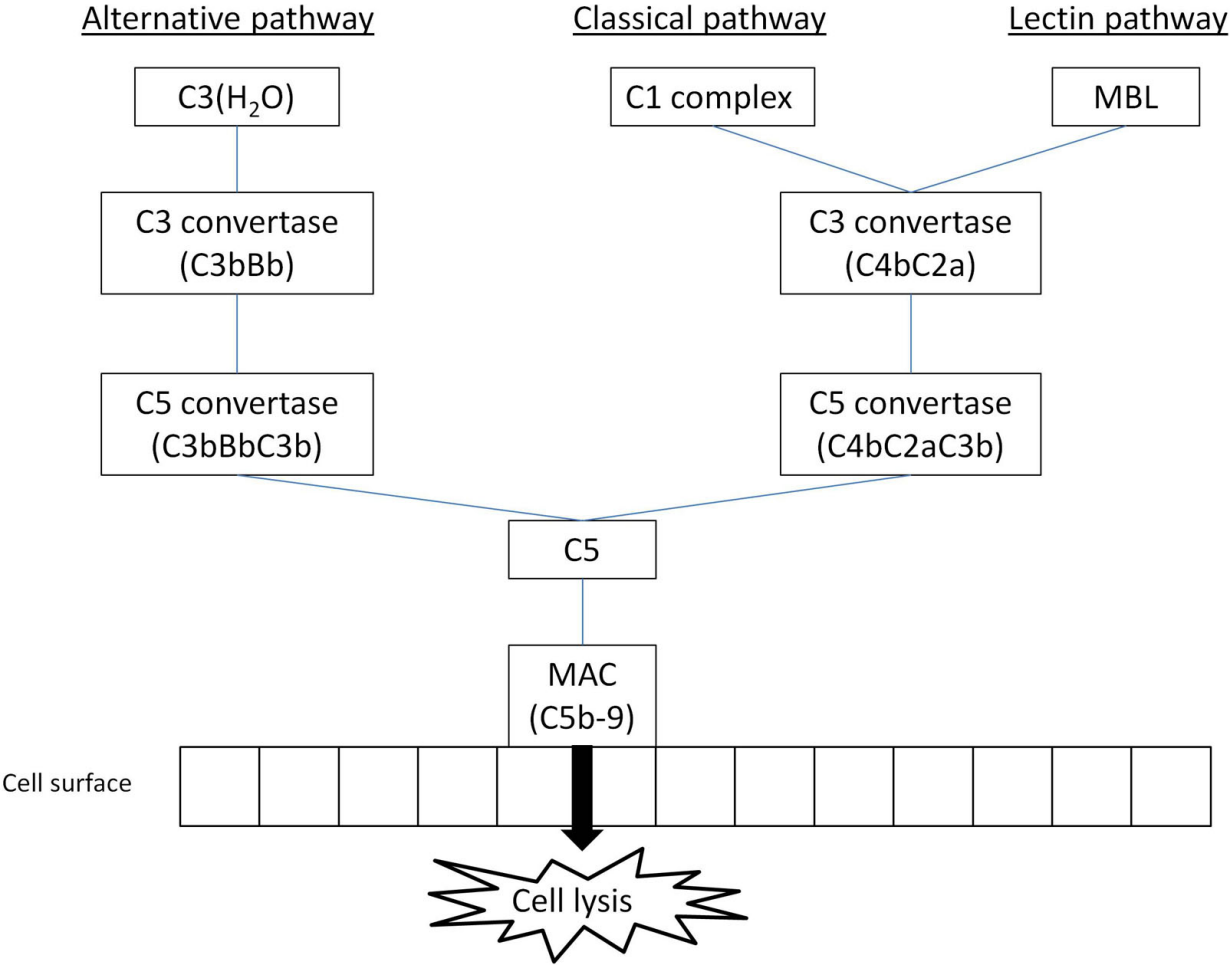
**The simplified overview of main complement activation pathways (MBL, mannose-binding lectin; MAC, membrane attack complex); three main activation pathways are recognized in the complement system, leading to anaphylatoxin release and MAC formation: classical and lectin pathways are reliant on special recognition molecules to recognize a target, alternative pathway is triggered spontaneously with host cells protected by regulator molecules**.

Complement activation can generally be achieved through one of three different pathways (Figure 1). All pathways require different stimuli to be triggered; the products of C5 cleavage and the formation of MAC are identical for all pathways (12). The classical pathway of the complement can be triggered by any structure that is recognized by C1q, the target recognition molecule for classical pathway. Mannose-binding lectin (MBL) and ficolins have the same function for lectin pathway (3). C1q can bind a wide variety of target structures, including antibodies IgM and IgG (certain isotypes), retroviral envelope proteins, or lipopolysaccharides, and is involved in immunological processes other than complement activation (13). MBL and ficolins are similar in structure to C1q to some extent; however, their C-terminal domains are different. These molecules are able to recognize and bind to saccharide patterns expressed on bacterial membranes and viruses (8). Lectin pathway can also be triggered by molecules found on the surface of dying cells (14).

In the classical pathway, the binding of C1q to its target molecule is followed by the recruitment of C1r and C1s components, forming a C1 complex (15). The newly formed C1 complex interacts with C4 and C2 complement components, which are expressed in their inactive forms, cleaving them and subsequently forming C4bC2a, a molecule with C3 convertase function (12). In lectin pathway, the target recognition molecules induce conformational changes in MBL-associated serine proteases (MASPs). This is followed by the assembly of C3 convertase, C4bC2a, similar to classical pathway (12).

The alternative pathway is different in a sense that it has no dedicated target recognition molecule. Instead, there is a constant process of low-level spontaneous hydrolysis (so-called “tick-over”) of thioester bond in C3 component, changing it into active molecule (16). This molecule can bind Factor B and induce its cleavage by Factor D. The resulting complex C3(H_2_O)Bb acts as a C3 convertase and is capable of cleaving native C3 molecules to C3a and C3b fragments (17). In order to prevent damage to healthy host cells, complement regulators are present both in plasma and on cell membrane, inactivating C3b molecules that bind to the cell surface. Further studies revealed a significant role of plasma protein properdin. This protein is produced by activated neutrophils and was proved to prevent the rapid spontaneous destabilization of C3bBb complex (18). It can also bind to C3b and prevent its cleavage by complement regulators (19). Properdin is sometimes described as the only known positive regulator of the complement cascade (20).

The C3 convertases for all pathways have the same function—the cleavage of C3 into C3a and C3b fragments. The C3b fragment can subsequently bind to C3 convertase, turning it into C5 convertase. The C5 convertases promote the complement cascade by cleaving C5 into C5a and C5b fragments (12). The composition of the MAC, which is formed on pathogen membrane by the C5b fragment created by C5 convertases and subsequent recruitment of C6, C7, C8, and several (10–18) molecules C9, is known for longer time (21). Its precise molecular structure is, however, still being examined and described in more detail (22).

Earlier studies had uncovered evidence of various agents capable of inducing C5 component activation without triggering one of the standard complement pathways, in a C3-independent manner (23). Later studies highlighted the relationship between complement and the coagulation system (24, 25) and, more importantly, suggested that C5 component can be cleaved by thrombin, generating C5a in the absence of traditional C5 convertases (26, 27). Recent study, however, questioned these conclusions, suggesting that thrombin influences C5a fragment generation indirectly and it is in fact plasmin that acts as a major C5 cleavage mediator (28).

### Anaphylatoxins and Their Function

Component anaphylatoxins, fragments C3a and C5a cleaved by their respective convertases, do not directly participate in the complement cascade progression. Instead, these small molecules are released and perform a number of immunomodulative functions. Anaphylatoxins possess significant pro-inflammatory functions, for example, leukocyte recruitment including neutrophils (29), mastocytes (30), and T lymphocytes (31), induction of oxygen radicals production in granulocytes (32, 33), induction of pro-inflammatory mediators production (34, 35), or blood vessel permeability increase (7). Apart from pro-inflammatory functions, studies showed that anaphylatoxins can also participate in tissue regeneration (36) and tissue fibrogenesis (37).

The complement activation is strongly associated with inflammatory processes. Recently, a new structure was identified in immune cells, a multiprotein complex called inflammasome. The inflammasome is important for proper induction and processing of IL-1β and IL-18, significant pro-inflammatory cytokines, *via* the activation of caspase-1 (38). Inflammasome assembly is induced by a variety of pattern recognition receptors, most notably Nod-like receptors, which also participate in its structure (39). Several studies focused on the link between complement activity and inflammasome activation, revealing that both C3a (40) and C5a (41, 42) anaphylatoxins are capable of influencing the IL-1 β production in immune cells. Newer research results have shown a novel mechanism of inflammasome activation in macrophages supported by MAC *via* the phagocytosis of opsonized particles (43). Since the complement system is an integral part of rejection mechanisms, it can be assumed that the complement-activated inflammasome formation could potentially influence the rejection processes, more research is, however, needed before any definitive conclusion is made.

### Complement Regulatory Proteins

To prevent the complement-mediated damage to healthy host cells caused by unchecked activation, regulatory proteins are expressed by the host cells on the cellular membranes or present in the plasma that can disrupt the function of convertases or their products (8). Some of the regulators are described as having decay-accelerating functions, which means they are able to directly influence and inhibit the structure and function of convertases. Membrane-bound decay-accelerating factor (DAF/CD55) causes the dissociation of Bb and C2a from their respective C3 convertases, while also preventing them from reassembling (44). Complement receptor 1 (CR1/CD35) also possesses decay-accelerating activity and is able to influence all three complement activation pathways through binding of C3b and C4b (45, 46) and functioning as a cofactor for Factor I, a protease capable of cleaving C3b and C4b components (47). The C4b-binding protein also works as a cofactor for Factor I, but is capable of targeting only C4b component and, therefore, plays a role only in classical and lectin pathways (48).

A number of regulators are aimed at alternative pathway products, since this pathway is triggered spontaneously and is, therefore, the most dangerous for host cells. Factor H plays a major role in the alternative pathway regulation; it functions as an inhibitor of Factor B binding to C3b, preventing the C3 convertase creation. Factor H was also shown to a have a role in C3b proteolytic cleavage as a cofactor for Factor I (49). Similarly, membrane cofactor protein (MCP/CD46) can act as a cofactor for Factor I, participating in proteolytic cleavage of both C3b and C4b (50).

## Complement in Renal Ischemia/Reperfusion Injury

The process of blood flow restoration after a period of its lack is common in organ transplantation. The ischemic injury to the renal tissue is a result of complex sequence of events, including renin synthesis, the shutdown of aerobic metabolism, and the generation of oxygen radicals (51). Together with other mediators such as pro-inflammatory cytokine release and complement activation, the environment becomes distinctly pro-inflammatory; leukocyte migration and activation are supported by these mediators (52). The expression of MHC class II molecules is higher both on endothelial and tubular epithelial cells during ischemia, supporting the T-cell response (53).

Both adaptive and innate immunity mechanisms are enhanced during ischemia/reperfusion injury. In our previous study with three renal graft biopsies describing thoroughly the ischemia/reperfusion process, the expression levels of several genes associated with renal allograft injury, i.e., cell proliferation and apoptosis, were shown to be upregulated during reperfusion (54). The complement activation is a significant marker of ongoing renal ischemia/reperfusion injury and has an influence on inflammatory processes. The C3 component expression is upregulated in the renal allograft (55) and the levels of systemic C3adesArg fragment (carboxypeptidase-cleaved C3a) released into circulation are increased (56). Interestingly, the main source of C3 component participating in ischemia/reperfusion injury was shown to be the renal allograft, with only minor role attributed to recipient’s circulating C3 (57).

Similarly, multiple studies have inquired into the role of C5 component during ischemia/reperfusion processes. The inhibition of C5 was shown to have the strong protective effect against inflammatory response in renal allograft, reducing significantly the influx of neutrophiles and suggesting that the chemokine production is dependent on complement activation during ischemia/reperfusion injury (58). Further study has confirmed the importance of C5a/C5aR interaction for neutrophile infiltration, while also hinting at the existence of different, neutrophile-independent functions of C5aR (59). The expression of C5aR was also found to be upregulated in deceased donor grafts, and the increased expression levels were associated with cold ischemia time and graft function (60). A study comparing the effects of C3a and C5a on the pathogenesis of ischemia/reperfusion injury has described C5a as having more significant impact (61). Since C5 component was shown to influence T-cell differentiation toward Th1 type (62), it is possible that not only innate but also adaptive immune mechanisms are influenced by C5 and its cleavage product. Recent study of the effects of C5a on human kidney tissue revealed the increase in pro-inflammatory cytokine production (IL-1β, IL-6, and IL-8) by the renal tissue linked to C5a stimulation (63).

Research comparing genome expression in renal allografts from living and deceased donors revealed that complement components were expressed at significantly higher levels before reperfusion in allografts from deceased donors (64). These results seem to be in agreement with earlier study that described increased activation of complement and other inflammatory mediators in kidneys recovered from rats with diagnosed brain death (65). Damman et al. (66) found the evidence of increased induction of C3 component in renal allografts in donors with diagnosed brain death, which was associated with decreased allograft functionality. Similar results were obtained from an experiment with murine cardiac allografts (67). Further study analyzing the complement activation markers in deceased kidney donors described a relationship between donor plasma levels of MAC and the observed graft functionality, with higher MAC levels correlating with acute rejection incidence (68). Newer, abovementioned study described an increase in systemic C5a levels in plasma of donors with diagnosed brain death, compared to living donors, although no correlation with graft function was found; the expression rates of C5aR in allograft were also increased in comparison to living donors (63).

Further studies have shown that the complement activation and inflammation associated with the ischemia/reperfusion injury also leads to an increased antibody production, which might influence the probability of late allograft failure (69). The T cell infiltration of the allograft is also supported by the activated complement, providing another potential negative influence for long-term outcome (70).

The significance of IgM antibodies for complement activation during ischemia/reperfusion injury was also addressed by researchers. Originally, it was considered to be a proof of classical pathway activation (71) and these findings were supported by further studies (72). Newer experiments, however, came with evidence that natural IgM antibody binds to MBL and triggers the lectin pathway (73), which has been further confirmed (74).

The question of participating complement activation pathways has been the subject of many studies and is still not fully resolved. The matter is complicated by the fact that the complement activity in ischemia/reperfusion injury appears to differ significantly depending on both the organ selected for study and the model used (75). For instance, Zhou et al. (76) showed that C4-deficient mice were not protected against renal ischemia/reperfusion injury, suggesting that classical pathway does not significantly participate. These results were further supported by a study that showed that gastrointestinal ischemia/reperfusion injury in mice is not dependent on C1q, a key component for classical pathway activation (77). However, newer study proved the presence of C1q in murine intestinal ischemia/reperfusion injury, casting doubt on the previously published results (78). The same study also confirmed the role of lectin pathway, while failing to find evidence of alternative pathway activation, which was originally supposed to play an important role (79). Recently, Miwa et al. (80), based on their murine DAF^−/−^CD59^−/−^ model, suggested that alternative pathway plays a significant role during early renal ischemia/reperfusion injury, with properdin having an important pathogenic role.

The role of lectin pathway in endothelium complement activation during oxidative stress was known for some time (81), and cytokeratin 1 was found to play a significant part in activation process on hypoxic endothelial cells (82). Subsequent experiments suggested that lectin pathway is connected to ischemia/reperfusion injury, with MBL deposition correlating with postischemic inflammation (83). Further studies stressed the importance of lectin pathway in renal (84) and gastrointestinal (77) ischemia/reperfusion injury in mice. Recent experiments suggested that lectin pathway indeed plays a significant role in ischemia/reperfusion injury, indicating that the activation of complement proceeds in a C4-independent manner, where MASP-2 plays a crucial role for complement activation, suggesting a possible novel lectin pathway activation alternative (85).

Based on the abovementioned experimental studies, recently several prospective trials in human kidney transplantation studies were initiated to investigate the effect of complement inhibition in the prevention of delayed graft function (NCT01919346 and NCT02145182). Anti-C5 monoclonal antibody (eculizumab) used as C5 complement component is widely implicated in renal ischemia/reperfusion injury. Experiments on animal models already showed a positive effect of terminal pathway blockade on graft function, suggesting a possible way of ischemia/reperfusion injury and delayed graft function prevention (86); however, very recent data from the PROTECT study (NCT02145182) on 286 kidney transplant recipients randomized to receive either eculizumab (1,200 mg prior reperfusion and 900 mg on first postoperative day) or placebo along with antithymocyte globulin failed to meet its primary end point (delayed graft function defined as dialysis within first posttransplant week), with the results from both arms not reaching statistical significance (87). Clearly, it is too premature to draw any other conclusion from the PROTECT study as secondary analysis has not been finished yet and no data were published beside initial press release.

## Complement in Posttransplant Thrombotic Microangiopathy (TMA)

The complement system is also highly involved in the TMA characterized by microvascular thrombosis with thrombocytopenia, hemolytic anemia, and red blood cell fragmentation. In the renal allograft, either *de novo* or recurrent posttransplant TMA can appear (88). Many cases of recurrent TMA have been linked to complement-associated gene mutations, such as CFH, CFI, MCP, C3, and others; however, similar to aHUS, gene mutation confirmation is not necessary for diagnosis as the genetical evaluation is far too complex and cannot necessarily prove complement-associated gene mutation while complement gene risk haplotypes are frequently found in the population. TMA occurs in a wide range of diseases other than originally described thrombotic thrombocytopenic purpura and HUS, such as autoimmune diseases, malignant hypertension, HCV infection, vascular rejection, and endothelial damage due to immunosuppressant, such as cyclosporine A, tacrolimus, and sirolimus, toxicity (89, 90). Many of those trigger complement (over)activity. TMA seems to be not a rare histological finding in kidney graft biopsies performed early after transplantation (91). With the advent of Luminex technology allowing detection of donor specific anti-HLA antibodies, many of TMA cases were shown to be associated with antibody-mediated rejection and C4d positivity. In a significant proportion of patients, however, the cause of TMA remains unclear. In many cases, TMA is associated with progressive graft dysfunction and graft loss despite the plasma exchange and immunosuppressive therapy. It is likely that in most cases, unrecognized complement genetic mutations risk haplotypes might be responsible for TMA development and subsequent graft failure. It was shown that even TMA associated with AMR may be associated with otherwise “silent” CFH mutation (92). Therefore, a blockage of terminal complement activity with anti C5 monoclonal antibody, eculizumab, has been introduced as a treatment of choice in recurrent TMA in kidney transplantation (93–95). Interestingly, many centers have been using eculizumab off-label also for *de novo* posttransplant TMA; however, it is premature to draw any conclusion as there is a lack of prospective studies and information from registries is still missing. Moreover, economical aspects of such therapy slow down clinical research in this interesting area.

## Complement and T-Cell-Mediated Rejection

While the complement system plays an important role in the innate immunity mechanisms, its role in the adaptive immunity functions is also significant. It is becoming apparent that the complement plays a significant role in T-cell-mediated rejection, influencing the proliferation and activity of T-cells participating in rejection (Table 1). The mechanisms of complement involvement are not yet fully understood, however, they are a subject of focused research. Despite the fact that majority of C3 complement component is expressed in liver, C3 can also be expressed locally in other tissues, including kidneys (96). Pratt et al. (97) showed that in the absence of locally synthesized C3 component, the acute rejection of the murine renal allograft was not as powerful and the T-cell response was significantly regulated. More recent study confirmed that T-cell alloresponse is affected by both C3a and C5a anaphylatoxins produced locally, by dendritic cells (98). Further experiments on both human and murine cells showed that the blockage of C3a and C5a interactions with their respective receptors in T-cells leads to regulatory T cells induction and stabilization (99).

**Table 1:** **Complement function in transplantation**.

Ischemia/reperfusion injury	Antibody-mediated rejection	T-cell-mediated rejection
Direct effect on cells, tubulointerstitial injury (membrane attack complex, MAC) (76) Pro-inflammatory stimulation (C3a, C5a) (59, 75)	Direct effect on cells (MAC) (135) Enhancement of T-cell response (MAC) (127) Pro-inflammatory stimulation (C3a, C5a) (135) The influence on IgG antibody production (CR1, CR2) (124)	Direct effect on T-cell proliferation and activity (C3a and C5a, MCP/CD46) (11, 110) The influence on T-cell differentiation and regulatory T cell induction (C3a, C5a, C4, C1q, DAF/CD55) (11, 107, 104, 105) The influence on monocyte infiltration (C5aR) (100)

An important role is also played by C3a and C5a receptors (C3aR and C5aR), which are expressed on numerous types of immune cells. Gueler et al. (100) described an increase in C5aR expression on infiltrating cells in renal biopsies in patients with acute cellular rejection, as compared to patients with histologically normal biopsies. Subsequent experiments with murine renal allografts showed the similar pattern and the use of C5aR antagonist pretreatment led to increased graft survival and the decrease in monocyte/macrophage infiltration. In the same year, another study revealed the connection between C3aR on the surface of dendritic cells and the attenuation of T-cell stimulation by cyclic adenosine monophosphate level alteration (101). Experiments performed on murine cells revealed that signaling *via* C3aR and C5aR on natural regulatory T-cells attenuates the FoxP3 expression, leading to diminished suppressive function and, subsequently, enhanced pro-inflammatory T-cell response (102).

As mentioned above, the complement can also influence the creation of regulatory T-cells (11). Recent study showed that in C3-deficient mice, the T-cell-mediated rejection of skin grafts was attenuated by the reduction of Th1/Th17 (i.e., pro-inflammatory) T-lymphocyte activity while upregulating the regulatory T-cell expression (103). Another study reported correlation of regulatory T-cell count and serum C4 levels in patients with systemic lupus erythematosus; according to the results of following *in vitro* experiment, the C4 component is able to influence the T-cell differentiation in favor of regulatory T-cells in the presence of dendritic cells (104). Similar effect was shown for C1q component, where macrophages that ingested C1q-opsonized apoptotic cells demonstrated the ability to affect the levels of Th1/Th17 T-cell proliferation, resulting in increased tendency toward regulatory T-cells (105).

The exact role of regulators of complement activation during T-cell-mediated rejection is not yet fully understood. Earlier studies showed that murine DAF homolog acts as a regulator of T-cell immune response in mice (106) and that DAF expressed on antigen-presenting cells has the ability to affect the T-cell differentiation by regulating the C5a component production (62). The experiments on murine cardiac allografts from DAF-deficient donors showed that DAF is able to regulate T-cell activity and to affect T-cell-mediated rejection (107). This effect was later confirmed in a study of graft-versus-host disease (108).

Recent study revealed the decrease in mRNA expression of CD59 and Crry (murine regulator of complement, functionally similar to MCP) in rat allografts compared to control group with syngeneic grafts. The same study also compared levels of MCP in patients’ clinical data and found a significant difference in 5-year renal graft survival, where the grafts of patients expressing high levels of MCP showed higher survival rates (109). MCP was also shown to be able to regulate the T-cell immune response by affecting the cytokine production of Th1 cells (110).

We recently described, in clinical settings, that T-cell-mediated acute rejection can occur even in patients with full C5 blockade while using eculizumab, suggesting that the role of C5 is not critical for T-cell-mediated rejection or, alternatively, other mechanisms are able to replace C5 functionally in such a case (111).

## Complement and Antibody-Mediated Rejection

While the main role of complement system during T-cell-mediated rejection is to regulate activity and differentiation of participating T-cells, in antibody-mediated rejection, the complement participates in more direct manner and its components can be found directly at the site of the antibody binding (112). Although the classical pathway is considered to be the primary mechanism of complement system activation during antibody-mediated rejection, experiments have also shown that the influence of MBL levels was found to be associated with rejection severity (113). Another study suggested connection between lectin pathway of activation and C4d deposition in kidney allografts, hinting at possible role of lectin pathway activated by H-ficolin in antibody-mediated rejection (114). The complement fragment C4d, created by C4 cleavage, has a specific significance for antibody-mediated rejection. Although being an inactive component, it remains bound at the site of C4 cleavage, acting as a marker of complement activation (115). The immunohistological assessments of renal allograft biopsy samples revealed that a deposition of C4d in interstitial capillaries can be linked to worse allograft outcomes and was suggested to be a marker of humoral response to the allograft (116). Further studies confirmed the importance of C4d as an independent predictive factor of long-term graft survival (117, 118). The presence of C4d deposits in allograft was included in Banff’ classification of antibody-mediated rejection as a criterium for positive identification in 2001, and in 2007 update the C4d scoring was further specified as a criterium for the identification of chronic antibody-mediated rejection (119, 120). Later studies, however, hinted at the existence of another, C4d-negative form of antibody-mediated rejection. A study comparing the protocol kidney biopsies from renal transplant recipients reached the conclusion that C4d presence cannot be considered an evidence of chronic antibody-mediated rejection (121), while the results of a study focused on endothelial gene expression in allografts suggested that only about 40% of renal transplants undergoing chronic antibody-mediated rejection are C4d-positive (122). Based on the results from studies questioning the usability of C4d positivity as criterium for chronic antibody-mediated rejection, the Banff classification was revised during the 2013 Banff Conference. The previous immunohistological evidence required for classification was changed into a requirement of the evidence of the antibody interaction with the endothelium. This new classification category includes C4d positivity, among other markers (120).

The classical pathway presents the dominant mechanism of complement activation during antibody-mediated rejection (123). The antibody production was found to be directly influenced through complement receptors CR1 and CR2 expressed on the surface of follicular dendritic cells (124), while antigens with C3d component attached were able to react more efficiently with B-cells *via* CR2, lowering significantly the amount of antigen needed to induce the antibody production (125). IgG and IgM antibodies bound on graft endothelium are capable of interacting directly with C1q, leading to the classical pathway activation of the complement, anaphylatoxin release, and MAC formation (126). In recent study, a novel view on complement activity in allograft injury was revealed; it was shown that the antibodies bound to the vascular endothelium lead to complement activation and the formation of MAC, which can subsequently trigger a non-canonical NF-κB signaling, resulting in pro-inflammatory reaction and T-cell response enhancement instead of cell lysis (127). While some studies showed the correlation between the presence of donor-specific antibodies (DSA) with complement-binding capability and decreased graft survival with higher rate of antibody-mediated rejection (128, 129), the results from different studies revealed no predictive value for these DSA concerning graft survival or rejection rates (130). Recent research uncovered the evidence of existence of polyreactive antibodies, capable of reacting to multiple different antigens, including HLA molecules (131); this discovery opposes the traditional view of antibody specificity to single target. The possible presence of these antibodies has been suggested to be related to the increased levels of autoantibody response in patients with chronic antibody-mediated rejection described earlier (132). Further study confirmed both the presence and the development of polyreactive antibodies in kidney transplants with chronic antibody-mediated rejection and also their ability to activate complement *via* classical pathway, contributing to C4d deposition in allograft tissue (133).

Several studies suggested a significant role of terminal complement activation during acute antibody-mediated rejection (134). Stegall et al. (135) later suggested that the role of complement in chronic antibody-mediated rejection could be, in contrast to its acute form, more oriented toward the chemotaxis and inflammation stimulation function. Therefore, terminal complement blockade was suggested to be an efficacious therapy in DSA-positive kidney transplantation. However, prospective randomized trial (NCT01399593) was ceased due to the lack of convincing efficacy in antibody-mediated rejection reduction, despite numerical difference between study and control groups (136). One of the possible explanations for these results is the involvement of complement-independent mechanisms of antibody-mediated rejection. Regarding this aspect, a study on mice revealed a significant role of NK-cells in chronic antibody-mediated rejection of heart transplants, since the depletion of these cells lowered the frequency and severity of rejection in allografts regardless of complement activation (137). These results tend to support conclusions of earlier study of transcripts expressed in renal biopsies obtained from DSA-positive patients. NK-cell activity was put in association with antibody-mediated rejection, regardless of C4d positivity (138). Further experiments confirmed the association of NK-cell transcripts with cases of antibody-mediated rejection and DSA positivity and also revealed NK-cell transcripts in some cases of early T-cell-mediated rejection, suggesting the possible participation of NKT-cells (139). The results of recent study on CCR5-deficient mouse model of renal transplant rejection provide further data supporting the link between antibody-mediated graft injury and failure and the activity of the NK-cells (140). Legris et al. (141) suggested that further study of peripheral NK-cell activity may provide additional data concerning the complement-independent antibody-mediated rejection mechanisms linked to NK-cells.

## Complement Targeting Therapies in Kidney Transplantation

Complement system plays a significant role in most cases of antibody-mediated rejection. Therefore, its components can be considered as potential therapeutic targets in attempts to prevent or treat such type of the rejection. Recently, the inhibition of several complement components has shown certain results in preventing the antibody-mediated rejection.

C1 inhibitor is a serine protease inhibitor that is capable of irreversibly blocking not only C1 complex components (142), as the name suggests, but also MASP-1 and MASP-2 (143). It is, therefore, an effective inhibitor of both classical and lectin pathway activation. Several studies with C1 inhibitor were already published. Earlier study on non-human primate model showed a potential for inhibition of acute antibody-mediated rejection with C1-inhibitor (144). Very recently, a double-blind study assessed the safety of C1-inhibitor in kidney transplant recipients and suggested the positive effect of this treatment regarding the C1q+HLA antibody levels reduction and antibody-mediated rejection occurrence (145). The possible use of complement inhibitor based on viral coat proteins is also a subject of ongoing research (146, 147).

Eculizumab is a humanized monoclonal antibody capable of blocking the cleavage of C5 complement component, thus preventing the release of anaphylatoxin C5a and the assembly of MAC (148). The C5 component participates in all three main pathways of complement activation; therefore, the blockade of its cleavage can have a positive influence on various medical conditions regardless of the active pathway. Eculizumab was shown to be an effective treatment for paroxysmal nocturnal hemoglobinuria (149) and aHUS including its recurrence in kidney transplantation (93, 150). Since the complement system is a prominent factor during antibody-mediated rejection, the effects of eculizumab were studied with regard to this condition as well. Stegall et al. (134) studied the effects of terminal complement inhibition on patients with a positive crossmatch and concluded that eculizumab is capable of decreasing the incidence of acute antibody-mediated rejection. Some success in treating the acute antibody-mediated rejection in renal allograft was also reported in several case reports (151–153). Extended follow-up study, however, showed that eculizumab treatment has no effect on chronic antibody-mediated rejection in recipients with significant amount of DSA (154). Moreover, there are known cases of C4d-negative antibody-mediated rejection where the use of eculizumab was ineffective (155). Similarly, the existence of IgM-mediated acute antibody-mediated rejection was described in patients treated with eculizumab (156). Therefore, C5 inhibition might be efficacious in the treatment of acute antibody-mediated rejection at least in some cases; however, these effects are lost in preventing chronic rejection development.

## Conclusion

The complement system is an intricate system of proteins participating in a wide range of processes connected to immune system. It possesses a significant influence and it is carefully regulated. The precise involvement of the complement system in the rejection processes is not yet fully understood, but it is becoming clear that it plays an important role in both T-cell-mediated and antibody-mediated organ rejection. Lately, new evidence is emerging suggesting that new, complement-independent mechanisms participate in transplant rejection as well. Recent uncertainty in particular complement proteins targeting therapies in kidney transplantation reflects the complexity and phylogenetical conservation of complement system.

## Author Contributions

MC wrote the paper. OV designed and wrote the paper.

## Conflict of Interest Statement

OV received a speaker fee from Alexion. MC declares no conflict of interest.
